# Assessing the implementation of a patient navigation intervention for colonoscopy screening

**DOI:** 10.1186/s12913-019-4601-4

**Published:** 2019-11-06

**Authors:** Amy DeGroff, Lindsay Gressard, Rebecca Glover-Kudon, Ketra Rice, Felicia Solomon Tharpe, Cam Escoffery, Joanne Gersten, Lynn Butterly

**Affiliations:** 1 Centers for Disease Control and Prevention, National Center for Chronic Disease Prevention and Health Promotion, Division of Cancer Prevention and Control, Program Services Branch, 4770 Buford Hwy, NE, MS K-76, Atlanta, GA 30341 USA; 2 Washington D.C., USA; 3 Department of Behavioral Sciences and Health Education Rollins School of Public Health, Emory University, 1518 Clifton Road, NE, 5th Floor, Atlanta, GA 30322 USA; 4 New Hampshire Colorectal Cancer Screening Program, Mary Hitchcock Memorial Hospital, Lebanon, NH USA; 5 Dartmouth-Hitchcock Medical Center, One Medical Center Drive, Lebanon, NH 03756 USA; 6 Department of Medicine, Geisel School of Medicine at Dartmouth, Lebanon, NH USA

**Keywords:** Cancer screening, Colonoscopy, Patient navigation, Disparate populations, Public health

## Abstract

**Background:**

A recent study demonstrated the effectiveness of the New Hampshire Colorectal Cancer Screening Program’s (NHCRCSP) patient navigation (PN) program. The PN intervention was delivered by telephone with navigators following a rigorous, six-topic protocol to support low-income patients to complete colonoscopy screening. We applied the RE-AIM (reach, effectiveness, adoption, implementation, maintenance) framework to examine implementation processes and consider potential scalability of this intervention.

**Methods:**

A mixed-methods evaluation study was conducted including 1) a quasi-experimental, retrospective, comparison group study examining program effectiveness, 2) secondary analysis of NHCRCSP program data, and 3) a case study. Data for all navigated patients scheduled and notified of their colonoscopy test date between July 1, 2012 and September 30, 2013 (*N* = 443) were analyzed. Researchers were provided in-depth call details for 50 patients randomly selected from the group of 443. The case study included review of program documents, observations of navigators, and interviews with 27 individuals including staff, patients, and other stakeholders.

**Results:**

Program reach was state-wide, with navigators serving patients from across the state. The program successfully recruited patients from the intended priority population who met the established age, income, and insurance eligibility guidelines. Analysis of the 443 NHCRCSP patients navigated during the study period demonstrated effectiveness with 97.3% completing colonoscopy, zero missed appointments (no-shows), and 0.7% late cancellations. Trained and supervised nurse navigators spent an average of 124.3 min delivering the six-topic PN protocol to patients. Navigators benefited from a real-time data system that allowed for patient tracking, communication across team members, and documentation of service delivery. Evaluators identified several factors supporting program maintenance including consistent funding support from CDC, a strong program infrastructure, and partnerships.

**Conclusions:**

Factors supporting implementation included funding for colonoscopies, use of registered nurses, a clinical champion, strong partnerships with primary care and endoscopy sites, fidelity to the PN protocol, significant intervention dose, and a real-time data system. Further study is needed to assess scalability to other locations.

## Background

The effectiveness of patient navigation (PN) in increasing breast, cervical, and colorectal cancer (CRC) screening among disparate populations has been empirically studied since the mid-1990s [1]. For increasing colorectal cancer screening, the focus of this paper, results of randomized and quasi-experimental trials of PN have largely been favorable [2–5].

The New Hampshire Colorectal Cancer Screening Program’s (NHCRCSP) was funded by the Centers for Disease Control and Prevention (CDC) from 2009 to 2015 to provide CRC screening and related support services, including PN. The NHCRCSP served a low income and ethnically diverse population. A recently completed effectiveness study of the NHCRCSP PN program demonstrated a significantly higher rate of colonoscopy completion among navigated patients compared to a similar group receiving usual care (96.2% vs. 69.3%, *p* < 0.001) [6]. Navigated patients were more than 11 times more likely to complete colonoscopy than those receiving usual care. Navigated patients also had no missed appointments (0.0% vs. 13.3%, p < 0.001), fewer cancellations within 24 h of the scheduled appointment (0.8% vs. 16.0%, *p* < 0.115), and more adequate bowel preparation compared to the usual care group (97.6% vs. 87.5%, *p* < 0.010) [6].

While these results are promising, decisions to replicate the intervention should be informed by looking beyond effectiveness to examine more practical aspects of implementation. Implementation refers to the content and conduct of a program, both what and how activities are delivered [7]. The “what”, the activities comprising the intervention, are detailed elsewhere [8, 9]. Examining the “how” of implementation is aided by applying RE-AIM, a multi-level framework for evaluating public health interventions in real-world settings [10, 11]. The purpose of this paper is to examine implementation processes (the “how”) by applying the RE-AIM framework and consider potential scalability of the intervention.

## Methods

A mixed-methods evaluation study was conducted including 1) a quasi-experimental, retrospective, comparison group study examining program effectiveness, 2) secondary analysis of NHCRCSP program data, and 3) a case study. This paper presents the methods and results for the second and third evaluation components only; the comparison group study methods and results are presented elsewhere [5]. This study was approved by CDC’s institutional review board and appropriate Dartmouth-Hitchcock committees approved the study protocol and methods.

### Program description

The NHCRCSP staff team included a program director, a medical director (gastroenterologist providing clinical oversight), two navigators, a secretary responsible for assisting in enrolling patients, and a data manager tasked with collecting and reporting program data to CDC. Two registered nurse navigators, one full-time and one quarter-time, delivered PN services telephonically to several hundred patients across the state annually whose screening costs were compensated by NHCRCSP with CDC funding. Patients were New Hampshire (NH) residents at average or increased risk for CRC, ages 50–64, < 250% federal poverty level, and uninsured or underinsured. NHCRCSP patients received their colonoscopy at one of 12 endoscopy sites throughout the state with which NHCRCSP contracted. Given limited resources, the most northern area of NH was not targeted given its small population. The CDC grant supported payment of relevant clinical costs, including colonoscopy. Navigators followed a prescribed protocol addressing six specified topics: (1) patient engagement, CRC screening education, and barrier assessment (e.g., transportation, fear); (2) bowel preparation (prep) education and barrier resolution; (3) prep review and readdressing barriers; (4) assessment of prep and confirmation of test day details; (5) day of colonoscopy check-in; and (6) follow-up and assurance of patients’ understanding of results and recommended follow-up [7]. The navigators documented interactions with patients in a real-time database system that also supported patient tracking and follow-up.

### Data sources

Data used in the evaluation included a de-identified, patient-level analytic dataset provided by the NHCRCSP and navigator call notes on a subset of patients. Case study data included program documents, results of a patient satisfaction survey, navigator observations, and interviews. All data sources and related analysis are described in detail below.

#### NHCRCSP patient-level program data

The analytic dataset included data for all NHCRCSP patients scheduled and notified of their colonoscopy test date between July 1, 2012 and September 30, 2013 (*N* = 443). To assess colonoscopy completion and other outcomes (e.g., missed appointments, bowel prep quality), data were assessed for a 12-month period from the notification date. Data variables included, among others: patient demographics, residence zip code, comorbidities and CRC personal and family history, number and length of calls (in five minute increments) per navigation topic, appointment no-shows and cancellations, bowel preparation quality, and colonoscopy completion. Basic descriptive statistical analysis of data for the 443 NHCRCSP patients navigated during the defined time period was conducted using SPSS Version 20 to assess patient demographics and outcomes. To determine program reach for this time period, patient zip codes were mapped using geographic software.

#### Navigator call notes

To examine time spent delivering PN and fidelity with the six-topic PN protocol, CDC researchers were provided de-identified, in-depth call details for 50 patients randomly selected from the larger group of 443. The call notes included the date of the call, length of time per navigation call, call topic, and whether the navigator successfully reached and spoke with the patient. A total of eight patients were excluded from these analyses. Three were excluded because their data included PN calls for additional colonoscopies performed outside of the study time period, thus differentiating them from the other patients. One was excluded because the patient moved out of state prior to completing the screening cycle. And four were excluded because they reported they were not interested in completing colonoscopy after learning more about the procedure, therefore, did not have the opportunity to receive all six call topics. This left a sample of 42 patients for analysis of delivery time and protocol fidelity.

Total PN delivery time was calculated based on the minutes allocated to any call related to the six-topic navigation protocol. PN delivery time includes time spent by navigators preparing for each call and time spent entering data following the call. To assess fidelity to the six-topic PN protocol, two CDC researchers independently reviewed the PN call details to determine whether the navigator successfully addressed each of the six topics. Researchers then identified any discrepancies in calculations. These differences were reviewed and discussed with a third researcher until agreements were achieved.

#### Program documents

The NHCRCSP team provided CDC researchers with multiple program documents (e.g., CDC grant application, NHCRCSP policy manual, PN protocol and related materials, data system specifications, zip codes for endoscopy sites). For analysis, documents were reviewed carefully and used to develop a logic model depicting the relationship between programmatic activities and outcomes and a patient flow diagram representing steps to completing colonoscopy including areas of interface with program staff and partners [9]. The logic model and patient flow diagram were reviewed and verified by NHCRCSP staff. Zip codes for the 12 endoscopy sites contracted by the program were mapped using geographic software.

#### Patient satisfaction survey results

CDC researchers were provided a summary report of satisfaction surveys completed by patients served by NHCRCSP from July 1, 2012 to June 30, 2013. A total of 405 patients were mailed a brief survey following program participation and were encouraged to voluntarily complete and return it. The survey was anonymous and completed by 235 patients (58% response rate). The patient satisfaction survey results were reviewed by the research team.

#### Navigator observations

Four CDC researchers each conducted 4 h (16 person-hours) of in-person observations of the navigators on May 13, 2014. Two researchers observed one navigator and two other researchers observed the second navigator. During the observation period, the navigators provided telephonic navigation to patients and made calls needed to facilitate service delivery (e.g., confirming appointment details with endoscopy staff). Researchers used a standardized, pilot-tested template [12] to record field notes, including navigator techniques. The template included a matrix for notating the type of PN activity (e.g., spoke directly with patient, spoke on behalf of patient) and the person or entity with whom the PN interacted (e.g., patient, family member, pharmacy staff). Researchers observed a variety of call types and were able to ask the navigator questions following each call to gain further insight on her approach. For confidentiality purposes, calls were one-way whereby researchers could only hear the navigator speaking.

To analyze observation data, field notes were more fully transcribed by observers into electronic format. The team of observers met to review field notes and the call matrices developed during the observations. Researchers focused on defining navigator characteristics (e.g., empathic, encouraging) and identifying techniques (e.g., active listening).

#### Stakeholder interviews

Purposeful sampling [13] was used to select interviewees. To collect varied perspectives about the program, the CDC researchers identified several roles for study participation including NHCRCSP non-navigator staff, navigators, endoscopists, practice administrators working at endoscopy sites, primary care providers who referred patients to the program, and partner organizations, including state public health officials. All interview guides are available as Additional files 1, 2, 3, 4, 5 and 6. The NHCRCSP team assisted in identifying and facilitating contact with potential interviewees. Overall, CDC researchers conducted 24 in-person interviews from May 13–15, 2014 with 27 participants (some interviews included more than one interviewee). Interviewees provided signed consent. Those interviewed included program staff (*n* = 6), program stakeholders (*n* = 9), gastroenterologists (*n* = 2), practice administrators at endoscopy sites (*n* = 5), and primary care providers (n = 5). The average length of the interviews was 45 min.

To conduct the interviews, the four CDC researchers split into two teams, each with a trained qualitative researcher with extensive interviewing experience. Within each team, one researcher led the interview and the second served as note taker. Interviewers followed open-ended interview guides specific to the interviewee role. Questions addressed the following topics: aspects of the PN model, experiences related to program implementation including reach, perceived challenges and facilitators to implementation, navigator characteristics and skills, perceptions of program effectiveness, partnerships, sustainability, and patient experiences.

CDC researchers conducted additional interviews by telephone with nine NHCRCSP patients from September 12–25, 2014. Patients were asked about their experience with the program and the navigator and interviews lasted, on average, 11 min. Across all interviews, saturation was achieved suggesting an adequate number of interview participants.

For analysis of interviews, a professional transcription service transcribed verbatim the digitally audio-recorded data. Researchers verified the accuracy of the transcription by re-listening to the interview audio and making needed corrections in a revised, final transcript. Immediately following vetting, the team created an analytic summary of each interview. Analysis of transcripts involved several iterative steps, consistent with inductive analytic techniques and constant comparative methods. Evaluators conducted initial, focused, and theoretical coding [14], in progression, each refined by memoing and testing for confirming or disconfirming evidence. To develop an analytic codebook, codes were developed and defined, then tested and refined on a data sample before being applied across all interviews. Once data were coded, data for individual codes were reviewed by stakeholder role. Analysis advanced through the development of matrices and inductive outlines to inform interpretation [15].

### RE-AIM analysis framework

The RE-AIM framework has five dimensions -- reach, efficacy, adoption, implementation, and maintenance [10, 11]. Reach measures the ability of the program to engage the priority population, while efficacy assesses the impact of the program on health outcomes. Adoption refers to uptake of the program by target organizations. Implementation reflects the consistent delivery of the program, and maintenance is the extent to which a program becomes institutionalized. Table 1 below provides the RE-AIM elements, their definitions, indicators, and data sources used for this study.

**Table 1:** RE-AIM Framework for Evaluating NHCRCSP Patient Navigation Program

Element	Definition	Indicator	Data Source
Reach	Ability to engage the priority population	Mode of navigator communication Geographic location of patients Methods of recruitment Number and characteristics of patients Challenges to reach	NHCRCSP program data Program documents Interviews
Efficacy	Program impact on health outcomes	Colonoscopy completion and other outcomes Stakeholder perception of efficacy	NHCRCSP program data Interviews
Adoption	Program uptake by target organizations	NHCRCSP Champion Number and geographic location of contracted endoscopy sites Partnerships Provider-site champions Adoption challenges	NHCRCSP program data Program documents Interviews
Implementation	Consistent delivery of program	Dose (time spent, number of days navigated) Fidelity with 6-topic protocol PN characteristics and training Monitoring and quality assessment Patient satisfaction Stakeholder perceptions of implementation Implementation challenges	NHCRCSP program data Program documents Interviews Navigator observations Navigator call notes
Maintenance	Extent program becomes institutionalizing	Resources Infrastructure Partnerships Staff turnover Challenges to maintenance	Program documents Interviews

## Results

### Reach

Two navigators (1.25 FTE) delivered the PN intervention to 443 patients during the 14-month study period. By delivering PN via telephone, navigators were able to reach patients across the state, aside from a small geographic area representing the least populated area in the state. Patients had access to a cell phone or landline. Patients were offered call cards with pre-paid minutes for their cell phones if needed. Patients received colonoscopy at one of 12 contracted endoscopy sites across the state.

Patients were recruited through provider referral (91.2%), self-referral (4.7%), and referral through another CDC-funded screening program, the National Breast and Cervical Cancer Early Detection Program (4.1%). Patients who self-referred included those who heard about the program through an outside source (e.g., family member, program website). NHCRCSP engaged federally qualified health clinics (FQHCs) as an important referral source given many FQHC patients are un- or under-insured.

The program successfully recruited patients from the intended priority population who met the established age, income, and insurance eligibility guidelines (Table 2). Among those served, 11.1% were Hispanic and 12.2% were non-White. Patients reported 21 different languages as their primary language, and nearly a fifth required translation services. NHCRCSP contracted with a language line with staff that could translate all PN calls. Additional details about the study population are reported elsewhere [6]. Table 3 provides qualitative data reflecting strategies and constraints around the program’s reach. These interview quotes reflect the efforts of the program to serve populations in high need geographic areas, but also that limited program resources constrained reach.

**Table 2:** NHCRCSP patient characteristics (*N* = 443)

Age (years)	
50–59	75.6%
60–64	24.4%
Sex	
Female	63.9%
Male	36.1%
Race	
White	81.7%
Asian	8.6%
Black/African-American	3.2%
Native Hawaiian/Pacific Islander	0.2%
American Indian/Alaskan Native	0.2%
Unknown	6.1%
Ethnicity	
Hispanic	11.1%
Non-Hispanic	86.9%
Unknown	2.0%
Education	
Less than high school graduate	15.8%
High school graduate or equivalent	37.3%
Some college or higher	44.2%
Unknown	2.7%
Primary Language	
English	79.7%
Spanish	9.7%
Other^a^	10.5%
Interpreter Needed	17.2%
Medical History	
Previously screened for colorectal cancer	29.4%
Family history of colorectal cancer	13.8%
Personal history of colorectal cancer	13.1%

^a^Other includes Nepali, Vietnamese, Portuguese, Indonesian, Mandarin, Arabic, Bosnian, Cantonese, Russian, Kurdish, Tagalog, Greek, Gujarati, Somali, French, Bengali, Krahn, and American Sign Language

**Table 3:** Selected Quotes Representing the Five RE-AIM Elements

Re-aim dimension	Qualitative data	Source
Reach	We literally sat down with a map of New Hampshire and looked at where the low income, uninsured, vulnerable minority populations were located, and we contracted with endoscopy sites, 12 of them, in those areas.	Medical Director
	I think because of [NHCRCSP’s] relationships with all the federally qualified health centers, they’re the key to always getting referrals.	Program Director
	Nothing’s perfect, but I guess the challenge is that the needs are probably greater than the program can meet.	
	I know they have some constraints with the number of patients that they can see.	
Efficacy	I’m not surprised that they’ve had success, knowing the [staff] people that are in this program and their dedication to that. That, with the backing of a lot of smart partners, I think that they were definitely set up to be successful, and they delivered on it.	Stakeholder
	Honestly, at the beginning I was not expecting the 100% success rate … I was really surprised to say it’s 98% of prepped success. Nobody has that. Nobody has that.	Stakeholder
	Oh, I think it’s the coaching, the navigator, a little bit of the pre-selection of people who are motivated to have it done. I mean, it’s not a procedure people are wanting to have done. So that it’s covered and they are able to have it done, and they know they need to, and it’s a population that’s plugged in to the medical community, I think they’re sort of motivated already to do it.	Endoscopist
	I would phrase it the other way to say that the people who call us seem, perhaps, to have a higher degree of motivation	NHCRCSP Staff
Adoption	You’ve got to have someone who’s passionate about it, and that comes across in everything [Medical Director] does. That really energizes partners, and so people know that she’s really true to the cause, really gets it; she understands the on the ground realities of certainly providers, but also partner organizations, and she, again, communicates that passion. She and [Program Director] are both very well connected.	Stakeholder
	So I just volunteered to be the “physician champion” for the sort of promotion of colorectal screening in our enterprise. So that’s how I got involved with [Senior PN] and her program and the other folks in our enterprise that have made up this committee to promote colorectal health.	Primary care physician
	Number one, they [NHCRCSP] had the Dartmouth brand and the Comprehensive Cancer Collaboration backing their work. So they chose already established, recognized partners on the ground in the state, so that helped. We helped with that as well, but Dartmouth and Comp Cancer already have credibility within the community, so that gave them a good jumping off point.	Stakeholder
	One thing we all struggle with is, because the program can only pay to a certain point, if someone has found something, whether they need cancer treatment or just surgery for some kind of lesion they found that’s not cancerous, but it’s something they couldn’t remove with a colonoscopy, what can be frustrating, depending on what healthcare system you work with, is … ok, now these people have no money. How are they going to get the care they need?	NHCRCSP Staff
Implementation	I think they’re doing an awesome job. Yup. And it was an excellent program that helped me out a lot, and that’s the bottom line.	Patient
	[Navigator] was really wonderful. She just, every time she would call, the first thing she would ask is ‘do you have any other questions or concerns, anything that you need to know?’ … the patient navigator was just a really nice touch to the program because it made me feel really at ease to know that I could get a hold of that person.	Patient
	Sometimes it’s difficult when they [patient] change their phone numbers, and you can’t reach them for an extended period of time. That becomes challenging. That becomes challenging to connect with that patient and to establish that rapport and that communication. I have learned that you always have to keep asking if that’s a current [phone] number.	NHCRCSP Staff
Maintenance	It’s very helpful, I think, to be located in an academic medical center [Dartmouth-Hitchcock]. It really has been helpful for a grant program like this, and the reason is that there’s tremendous support. Dartmouth already has, as any academic institution will have, reach into the community on its own to some degree. There is expertise everywhere for whatever you want … So it really does provide access if you have a problem with anything, whether it be medical or statistical or research or whatever it is.	NHCRCSP Staff
	We would not be where we are [without the data system] with any of this, patient navigation, it would not be supported … And then when it comes to the CCDE [data reporting] time, we’re done. So that is like the daily reports that they can run to see who they need to call today, what type of call … We have so many reports, now. So the database, it’s like a live other person for us. I mean, really, it would be a whole other person.	NHCRCSP Staff
	So they’re [American Cancer Society] a key, key partner, but we’ve made other partners, like Anthem has been a great partner. We knew we had to meet with them because they have a family practice doctor who is their medical director. So he gets us in places we wouldn’t be able to get into without him. I mean, wherever we can find a partner, we’ll take a partner.	NHCRCSP Staff
	I’m so lucky with the two people [navigators] that we have right now … If you hire the wrong person, it’s a challenge.	NHCRCSP Staff

### Efficacy

Results of a comparison group study examining outcomes among NHCRCSP patients and a control group of similar patients at one of the 12 endoscopy sites have been reported elsewhere [6]. Analysis of the 443 NHCRCSP patients navigated during the study period show that 97.3% completed colonoscopy. Additionally, there were zero missed appointments (no-shows) and 0.7% late cancellations (Table 4). Finally, 99.1% of patients had adequate bowel preparation quality, 100% had their results communicated to them and their primary care providers, and 100% had a recommended screening interval consistent with clinical guidelines. Qualitative data with stakeholder perceptions of efficacy are included in Table 3.

**Table 4:** Outcomes for NHCRCSP navigated patients, July 1, 2012 to September 30, 2013 (*N* = 443)

Outcome	Percent
Colonoscopy completed	97.3%
Adequate bowel preparation quality	99.1%
Missed appointment/no-show	0.0%
Cancellation < 24 h prior to appointment	0.7%
Results communicated to patient	100.0%
Results communicated to primary care provider	100.0%
Recommended rescreening interval consistent with clinical guidelines	100.0%

### Adoption

The medical director, a well-respected gastroenterologist in New Hampshire, acted as the program champion, promoting the program among fellow endoscopists, and was a key component to this successful program. The medical director was viewed as a strong advocate for increasing high quality colonoscopy screening and had credibility with colleagues throughout the state. As a result, twelve endoscopy sites, geographically dispersed across the state, adopted the program and were contracted by NHCRCSP. Navigators also recruited local champions working within endoscopic and primary care practices who adopted the program as their own and promoted it within their settings.

Partnerships were critical to program adoption and the medical and program directors had extensive relationships in the cancer community, drawing on them to engage primary care practices and other stakeholders such as the American Cancer Society. In addition, a pharmaceutical chain with locations throughout the state and accessible to NHCRCSP patients was enlisted to provide patients bowel prep materials that were reimbursed by NHCRCSP. A potential challenge for adoption was the lack of resources to pay for cancer treatment among persons diagnosed through NHCRCSP, given the population was uninsured and CDC resources could not be used to pay for cancer treatment. However, prior to implementation, program staff obtained commitments from directors of health systems associated with all the endoscopy sites for free cancer care for the few colorectal cancers anticipated to be diagnosed through the screening program. This commitment was readily given and adhered to for patients requiring cancer care. While the issue of the cost of cancer care was mentioned in interviews, interviewees may have been unaware of commitments made by their leadership, and researchers did not find strong evidence suggesting it was a significant barrier. Table 3 includes qualitative data addressing program adoption.

### Implementation

**Fig. 1:**
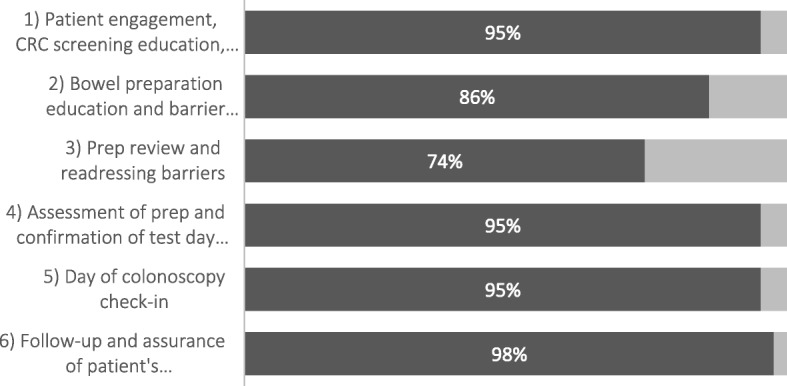
Percent of NHCRCSP patients who received each PN topic by telephone (*N* = 42)

Navigators spent an average of 124.3 min delivering the six-topic PN protocol to patients (range: 40–240 min). Protocol fidelity with four of the six topics was 95% or greater adherence (Fig. 1). The lowest level of fidelity (74% adherence) was observed for topic 3, prep review and readdressing barriers.

Based on observations of navigators, delivery of navigation was of high quality and in accordance with written protocols. Because navigators were nurses, they had the clinical expertise and capacity needed to understand and address medical issues related to colonoscopy and psychosocial assessment skills to effectively address patient barriers. Navigators received eight weeks of training that included instruction, coaching, and supervised practice. Training addressed CRC screening and surveillance, colonoscopy, other CRC screening tests, the PN protocol, cultural issues, and motivational interviewing, among other topics. Additionally, navigators received ongoing mentoring from senior clinicians throughout the program.

Observations revealed navigators who were professional, respectful, empathic, knowledgeable, and encouraging. One implementation challenge navigators faced was tracking patients who were sometimes transient. When possible, navigators gathered numerous phone numbers for patients so that if they could not be reached at their own cell or land lines, the navigator could contact relatives or friends to help reach the patients.

Navigators benefited from a real-time data system that allowed for patient tracking, communication across team members, and documentation of service delivery. Navigators used the system to closely track patients and monitor patient care, including colonoscopy completion. The medical director and program director regularly monitored PN service delivery and patient outcomes. Managers met weekly with navigators to review monitoring reports, discuss individual patient cases, and provide mentoring and support. This system of data collection and review was integral to NHCRCSP’s quality assurance efforts.

Based on survey data, patients had high satisfaction with the program and their navigation experience (Table 5). In addition, stakeholders perceived the program as of high quality, as evidenced by selected quotes in Table 3.

**Table 5:** NHCRCSP patient satisfaction survey results, July 1, 2012 through June 30, 2013 (*N* = 235)

Survey Item	% Yes	% Unsure	% No
The NHCRCSP intake and enrollment staff was helpful and courteous during the enrollment process.	100%	0%	0%
My Patient Navigator (nurse who called me several times) explained the colonoscopy so that I could understand the test.	100%	0%	0%
My Patient Navigator explained the instructions for drinking the prep for the colonoscopy so that I could understand them.	99.5%	0.5%	0%
I would recommend this program to a friend or family member.	99.5%	0.5%	0%
All of my questions were answered.	99%	1%	0%

### Maintenance

Evaluators identified several factors supporting program maintenance including consistent funding support from CDC, a strong program infrastructure (e.g., based in an academic medical center, Dartmouth-Hitchcock, contracts in place with endoscopy sites and primary care providers, effective data system, program manual), and partnerships (e.g., primary care providers, endoscopists, pharmacies, laboratories, transportation services, translation services). Staff turnover was also low. The medical director and program director were constant for the full CDC grant period. The data manager and secretary exhibited deep understanding and passion for the program. Navigators originally hired for NHCRCSP were replaced with different nurse navigators prior to our study. Those two navigators remained with the program through the study’s end (Table 3).

## Discussion

This study applied the RE-AIM framework to examine implementation processes of a PN intervention found effective in improving outcomes, including colonoscopy completion.

Results identified multiple implementation factors that likely contributed to intervention effectiveness including using telephonic navigation to extend reach and providing payment for colonoscopy in order to serve a low-income population. A gastroenterologist serving as a program champion, strong partnerships with primary care clinics and endoscopic sites, and a commitment of charity care for those diagnosed with cancer all supported program adoption. Implementation was aided by fidelity to a rigorous protocol, significant intervention dose, use of well-trained registered nurses with regular clinical supervision, and a real time data system for patient tracking and program monitoring. Maintenance of the intervention was possible given consistent program resources, a strong infrastructure, and low staff turnover.

We also found that applying the RE-AIM framework allowed examination of dimensions of implementation often ignored but essential to inform potential transferability and scalability. While calls for comprehensive evaluations addressing dimensions beyond effectiveness and efficacy have increased [16, 17], comprehensive assessments such as the mixed methods study presented here remain rare. Below, we consider the transferability of the NHCRCSP PN intervention based on each RE-AIM element.

Important considerations of reach for adopting this intervention in new settings include the ability to recruit the identified priority population and contact them by phone, as well as the availability of a payment source for colonoscopy and, if necessary, cancer treatment. For programs without a ready source of patients, partnerships with health systems serving the priority population will be needed. Also, in this study, while NH is largely rural, limited resources led NHCRCSP to prioritize more populated areas and, therefore, the program was unable to include those in the least populated parts of the state. Others have identified challenges in recruiting rural populations for cancer screening programs and recommend targeted outreach strategies [18, 19]. Next, replication of the intervention demands that the priority population has adequate phone access. Other navigation studies have documented significant challenges in reaching low-income populations by phone [20], including persons not answering calls, not having a phone, or not being able to afford adequate ‘minutes’ to respond to calls. The NHCRCSP successfully addressed these issues by confirming times that patients would be available for calls, collecting secondary phone numbers of friends or family, and providing paid phone minutes when needed. Finally, lack of insurance has repeatedly been identified as a barrier to colorectal cancer screening [21, 22]. In this study, CDC grant resources were available to pay for colonoscopy for an otherwise under- and uninsured population. Future adopters of the intervention may need to focus on insured populations if resources for colonoscopy are not otherwise available.

Along with results of the comparison study previously reported [6], data from this study provide additional evidence of a highly effective intervention, with over 97% of low-income patients completing colonoscopy. Further, this particular PN intervention positively affected several additional outcomes, some well-known and others that rarely or never have been reported in the literature (i.e., adequate bowel preparation, missed appointments, late cancellations, communication of results to patients and primary care providers, and the consistency of recommended rescreening interval with clinical guidelines). Some of these, such as missed appointments and late cancellations, have important financial implications for endoscopy sites. Additionally, good bowel prep quality reduces cost and discomfort for patients who avoid repeat screenings. Improved guidelines for re-screening can also reduce medical costs and patient risks associated with over-screening. Of note, a recently published analysis found the NHCRCSP PN intervention to be cost-effective in increasing colonoscopy screening, providing further evidence of this intervention’s value to public health [23].

While a few stakeholders suggested NHCRCSP patients may have been especially motivated to complete colonoscopy, data assessing patient motivation were not collected, and, therefore, this hypothesis is speculative. In our study, colonoscopy costs were covered for both the NHCRCSP patients and the comparison patients, eliminating cost as a distinguishing factor. Results might be different if patients for whom the cost of colonoscopy is not covered are compared to patients for whom it is; it is reasonable to assume the latter group might be more motivated. At the time of enrollment, patients did agree to participate in a colonoscopy, suggesting that they were amenable to the procedure. Given the complexity of colonoscopy [24], programs may benefit from assessing patient readiness and commitment to following through on colonoscopy early in the process, as was done early in the NHCRCSP protocol. Studies indicate that patients have preferences for particular CRC screening test types [25, 26]; therefore, colonoscopy is not necessarily the best choice for all average-risk patients, for whom screening test options are appropriate.

Adoption of the NHCRCSP PN intervention was facilitated by program champions and multiple, strong partnerships. There is evidence that champions contribute to the effectiveness of public health programs [27]. The primary champion for the NHCRCSP was a gastroenterologist who had the respect and credibility to recruit endoscopy and primary care sites. In another analysis, we found that a strong program champion with clinical expertise was an important component of this PN intervention [9]. Champions at the facility-level (e.g., primary care offices, endoscopy sites) also were recognized by interviewees as important to program adoption. In regard to partnerships, public health recognizes their importance to achieving public health outcomes [28], and previous research identified them as an important facilitator of CRC screening programs [29]. Scaling of this intervention, then, requires identification of strong program champions and appropriate partnerships.

A potential barrier to adoption if serving an under- or uninsured population is the availability of treatment resources for patients diagnosed with cancer. Other CDC-funded cancer screening programs have identified this as a significant challenge [30]. In such instances, programs can follow NHCRCSP’s example and try and secure resources or commitments (e.g., charity care) for cancer treatment in advance. Fortunately, a compelling business case can be made to encourage healthcare organizations to provide free cancer care in support of CRC screening programs, since increased screening will decrease incidence of CRC and correspondingly decrease the number of patients requiring free colorectal cancer care, which is extremely expensive, from those healthcare organizations.

In regard to implementation, results suggest that the PN intervention was delivered with fidelity to the PN protocol and with significant dose (i.e., minutes of delivery time). These factors have been shown to contribute to positive health outcomes [27]. Use of registered nurses as navigators who were given extensive training, clinical supervision, and management support also contributed to strong implementation. The program was found also to be data-driven, with staff committed to data monitoring and use based on a real-time data system. Program monitoring has previously been identified as an independent predictor for effectiveness [27] and is an important tool to improve clinical quality. These factors (i.e., fidelity, dose, training, supervision, management, data monitoring and use) can be explicitly addressed in any replication and/or scale up of the intervention. One implementation challenge experienced by navigators involved tracking some patients given their transience and/or lack of access to a consistent phone number. Collecting multiple contact numbers for patients as was done by NHCRCSP navigators and persistence in patient tracking efforts is therefore indicated in future replications of the intervention.

Maintenance of the PN intervention was enhanced through availability of consistent program resources, a strong infrastructure, and limited staff turnover. This suggests that replication of the intervention will benefit from well documented program materials and a carefully vetted and selected staff. CDC, in collaboration with NHCRCSP, has recently produced a replication manual of the PN intervention [8]. The National Cancer Institute has also recognized the PN intervention as a Research Tested Intervention Program (RTIPs) and the replication manual is available through their RTIPs website (https://rtips.cancer.gov/rtips/index.do). The manual details all facets of the intervention including the 6-topic protocol. Appendices include sample job descriptions for the navigators, the training curriculum, referral forms, and other needed resources to adopt the intervention. This resource is intended to aid future adoption and maintenance of the intervention. The sustainability of the intervention was not evaluated, and, therefore, it is not clear whether the intervention could be maintained without CDC or other resources.

Overall, evaluation results, based on the RE-AIM framework, are positive and support further study of the NHCRCSP PN intervention in new settings. Given that this was a relatively small program in a single state (the program served about 2000 patients throughout the state over 5–6 years), additional research is needed to examine scalability and explore generalizability. Scalability is defined as “the ability of a health intervention shown to be efficacious on a small scale and or under controlled conditions to be expanded under real world conditions to reach a greater proportion of the eligible population, while retaining effectiveness” [31]. Future evaluations of the NHCRCSP model in new settings will inform scalability of the intervention.

Limitations of the current study should be noted. First, the retrospective design of the effectiveness evaluation render results that are not generalizable. Second, evaluators had limited data (i.e., sample of cases) for calculating some process measures, including time spent delivering the intervention and fidelity with the six-topic PN protocol. Third, patient satisfaction surveys were not completed by all participants and it may be that those patients more satisfied chose to complete and return them. However, nearly 60% of patients completed the survey, which is a relatively high response rate.

## Conclusions

This evaluation of NHCRCSP PN intervention provides robust evidence supporting further investigation to explore scalability. Factors supporting implementation included funding for colonoscopies, use of registered nurses, a clinical champion, strong partnerships with primary care and endoscopy sites, fidelity to the PN protocol, significant intervention dose, and a real-time data system. For public health practitioners considering use of the NHCRCSP PN model, results presented here offer rich information to help assess whether the intervention is a good fit for their unique context and whether they have the capacity to adopt and adapt it. A replication manual for the intervention has been developed and evaluations are underway to assess the intervention in new settings to examine adaptation.

## Supplementary information

**Additional file 1.** Interview guide used with NHCRCSP non-navigator staff.

**Additional file 2.** Interview guide used with NHCRCSP navigators.

**Additional file 3.** Interview guide used with endoscopists involved in the NHCRCSP.

**Additional file 4.** Interview guide used with practice administrators at endoscopy sites involved in the NHCRCSP.

**Additional file 5.** Interview guides used with primary care providers involved in the NHCRCSP.

**Additional file 6.** Interview guides used with partner organization staff involved in the NHCRCSP.

## Data Availability

The data generated and analyzed during the study are not publicly available (no public access) but are available from the corresponding author on reasonable request.

## Abbreviations

CDC: Centers for Disease Control and Prevention
CRC: colorectal cancer
FQHC: Federally qualified health clinic
NH: New Hampshire
NHCRCSP: New Hampshire Colorectal Cancer Screening Program
PN: patient navigation
RE-AIM: reach, efficacy, adoption, implementation, maintenance
