# Supplementary material for: Assessing the implementation of a patient navigation intervention for colonoscopy screening
Source: BMC Health Serv Res. 2019 Nov 6;19:803. doi: 10.1186/s12913-019-4601-4 (PMC6833190; doi:10.1186/s12913-019-4601-4)
Supplement: Supplementary file 1 — Additional file 1. Interview guide used with NHCRCSP non-navigator staff. [file 12913_2019_4601_MOESM1_ESM.docx]

**Interview Guide for GRANTEE STAFF**

**Introduction and Informed Consent Statement**

Hi. My name is __________ with the Centers for Disease Control and Prevention. Thank you for giving us this opportunity to discuss your experiences with New Hampshire’s Patient Navigation for Colonoscopy Program. This should take no more than [*60-90 minutes, depending on role]* of your time, and we’ll do our best to stay on track. Before we begin, let me explain the purpose of the study and your rights as a participant. Did you receive the informed consent form in the mail *[or by e-mail]*?

[*For in-person interviews, give one copy of the Informed Consent Form to the participant. Read the consent form as the participant follows along. Ask the participant if he/she has any questions about the study. After questions are answered, ask whether the participant would like to participate in the interview and, if so, ask the participant to sign the form. Next, ask if the participant gives permission to turn on the audio recorder and, if so, ask the participant to mark “Yes” where indicated. Collect the signed Informed Consent Form and give the participant a clean copy for his/her records. Proceed with the interview.]*

[*For telephone interviews, continue reading]*

In partnership with New Hampshire’s Colorectal Cancer Screening Program, managed by Dartmouth-Hitchcock Medical Center, the Centers for Disease Control and Prevention (CDC), Division of Cancer Prevention and Control, is conducting an evaluation of program impact. Simply stated, we want to understand how patient navigation can improve cancer screening through colonoscopy.

Let’s go over a few key points:

- This interview is not meant to evaluate you;
- Rather, it is meant to learn from you how patient navigation affects colorectal cancer screening. There are no right or wrong answers.
- There are no expected risks to participation. But you may find it awkward or uncomfortable to answer questions about your experience. As a CRCSP staff member, it is also possible that your employer could use your responses against you. To prevent this, we will not give names or positions with direct quotes in any reports. We will also allow you to review your transcript and omit any information. We will not share your transcripts or responses with any other staff.
- There are no direct benefits to participating in this interview. But you may find it valuable to reflect on your experience.

We are interviewing many people in different roles to get a more complete picture of the program. You are the expert on your experience, and your opinions and thoughts are very important.

This interview is strictly confidential; meaning, information that identifies you will not be shared with anyone except our evaluation project team. We will never report your comments by name in any report.

Your participation is voluntary. You may choose not to answer some of the questions or you may choose not to participate without penalty. You can stop the interview at any time for any reason. If you would like more information about the study or if you would like to withdraw from the study, you may contact the Principal Investigator, Dr. Amy DeGroff at 770-488-2415. If you have questions about your rights as a participant in this study, please contact CDC/ATSDR’s Acting Deputy Associate Director for Science at 1-800-584-8814. Leave a message with your name, phone number, and refer to CDC protocol #6569 and someone will call you back.

We would like to audiotape our conversation to assist with note taking and to make sure we accurately capture our discussion. Transcripts of audio files will be labeled with pseudonyms or fake names, and audio files and notes will be destroyed when the project is finished.

**Do you have any questions before we get started**? [ADDRESS ANY QUESTIONS AND THEN BEGIN.]

**Before we start our discussion, I would like to get verbal consent to proceed. Do you agree to participate in this interview?**

- Yes 🡪 Thank you. I confirm that you are willing to answer the questions in this discussion and will note your verbal consent. We would also like to record the conversation to make sure we don’t miss anything.
- No 🡪 *Thank participant for his or her time and end conversation.*

**Do I have your permission to turn on the audio recorder?**

- Yes 🡪 Thank you. *Turn on recorder.*
- No 🡪 Thank you. I will refrain from recording the session.

Let’s start with a few questions about your role with CRCSP. We will focus on the PN component, specifically.

1. Please describe your role with CRCSP and with the patient navigation component.

Probe: Please describe ways you interact with the navigators. How does your role support patient navigation?

Probe: What skills do you use to support patient navigation or the navigators?

Now, I have a few questions about the program model….

1. **Medical Director/Program Director**: Think back to the very beginning. How did you know (or strongly suspect) that statewide, telephonic navigation by nurses would work well here?

Probe: Was this model based on an existing program or a particular theory?

Probe: Were other models considered as well? Why were these other models considered a good (or poor) fit?

1. How easy or difficult has it been to implement the patient navigation component of CRCSP? Why has it been so easy or difficult?
2. **Medical Director/PD**: What type of background, skills, and training is necessary for navigators?

Probe: Please tell me about your decision to use nurse navigators rather than community health workers, for example. Is clinical expertise important? Please explain why or why not.

Probe: In recruiting navigators, what characteristics were you looking for the most?

**Data Manager/Clinical Secretary**: What are special characteristics of your two navigators?

Probe: what makes them so good at what they do?

1. Please tell me about some of the major challenges in implementing patient navigation since the program began.

Probe: Thinking back, from start-up until now, can you recall key developmental milestones along the way? That is, what were some key struggles or accomplishments during start-up, early implementation of PN, and more mature implementation?

1. Who did you imagine serving in the beginning, in terms of eligible patients? Are you reaching your intended target population? Are there hard to reach populations that aren’t being served by the program? If so, please describe them.
2. Please describe how “patient empowerment” became a central philosophy for delivering services under this model. How has that framework shaped how patients are enrolled and how patient navigation is delivered?

Probe: How is boundary setting affected? For example, how does a patient empowerment approach affect which patients come into the program, what happens to them once they’re enrolled, and how far navigators can go to provide services?

1. What is the significance of this program to the state, your organization, to medical practice, and to public health?

Probe: how does this program advance high-quality colonoscopy & why should the general public care about this?

Probe: how does this program advance population-based screening & why should the general public care about this?

Probe: How does this program reduce health disparities?

1. What has surprised you about this program? Has it had any spillover benefit or unintended consequences relative to original design?
2. What is the biggest lesson you’ve learned, based on your involvement with the program?

**Partners, Systems and Teamwork**

Next, we’ll turn to partners, systems, and teamwork….

1. The patient navigation component of this program has a lot of moving parts (such as hiring & training navigators, establishing networks, database development, establishing intake/eligibility procedures, providing patient education/navigation, communication with PCPs, GIs, etc.). What parts must function well to keep all the other parts humming along?
2. Please tell me how this team works together to accomplish its goals.

Probe: whom do you interact with the most & why (i.e., on what types of tasks)?

Probe: how does the team communicate and coordinate various functions?

1. How important is Dartmouth-Hitchcock Medical Center to this program in terms of support and reputation?
2. Please tell me how patient navigation is designed to integrate with existing networks and partnerships? What part of these network/systems connections were easier to foster? What were the trickier ones?
3. Describe how you work with your medical advisory board (MAB) and other stakeholders. How, if at all, do they help champion the program?

Next, I have a few questions about sustainability and potentially replicating this program elsewhere..

**Transferability & Sustainability**

1. How can the PN component be maintained to achieve its intended outcomes over time? Could this be sustained without funding? How will ACA implementation affect sustainability?
2. If this program were to be replicated in another setting, what would that program need to have in place to be successful?
3. Please talk about the affordability of the patient navigation component---would other settings find this program affordable? If a program had fewer resources, what are the most essential elements to keep?
4. How important is the medical model to providing patient navigation?

Probe: If a program wanted to replicate CRCSP and didn’t have a medical director champion, like Dr. Lynn Butterly, how would it work?

1. Are there plans to replicate or adapt the model elsewhere? To what extent can the practice be applied and/or adapted across a variety of contexts (e.g., GI clinics)? What contexts seem well suited? What contexts do not?

Lastly…..

1. Is there anything I didn’t ask about that you feel is important to mention?

**Thank you so much for your time today. Your insights will help us to better understand the patient navigation program.**

**[stop audio recorder]**
