# Supplementary material for: Assessing the implementation of a patient navigation intervention for colonoscopy screening
Source: BMC Health Serv Res. 2019 Nov 6;19:803. doi: 10.1186/s12913-019-4601-4 (PMC6833190; doi:10.1186/s12913-019-4601-4)
Supplement: Supplementary file 2 — Additional file 2. Interview guide used with NHCRCSP navigators. [file 12913_2019_4601_MOESM2_ESM.docx]

**Interview Guide for PATIENT NAVIGATORS**

**Introduction and Informed Consent Statement**

Hi. My name is __________ with the Centers for Disease Control and Prevention. Thank you for giving us this opportunity to discuss your experiences with New Hampshire’s Patient Navigation for Colonoscopy Program. This should take no more than *90 minutes* of your time, and we’ll do our best to stay on track. Before we begin, let me explain the purpose of the study and your rights as a participant. Did you receive the informed consent form in the mail *[or by e-mail]*?

[*For in-person interviews, give one copy of the Informed Consent Form to the participant. Read the consent form as the participant follows along. Ask the participant if he/she has any questions about the study. After questions are answered, ask whether the participant would like to participate in the interview and, if so, ask the participant to sign the form. Next, ask if the participant gives permission to turn on the audio recorder and, if so, ask the participant to mark “Yes” where indicated. Collect the signed Informed Consent Form and give the participant a clean copy for his/her records. Proceed with the interview.]*

[*For telephone interviews, continue reading]*

In partnership with New Hampshire’s Colorectal Cancer Screening Program, managed by Dartmouth-Hitchcock Medical Center, the Centers for Disease Control and Prevention (CDC), Division of Cancer Prevention and Control, is conducting an evaluation of program impact. Simply stated, we want to understand how patient navigation can improve cancer screening through colonoscopy.

Let’s go over a few key points:

- This interview is not meant to evaluate you;
- Rather, it is meant to learn from you how patient navigation affects colorectal cancer screening. There are no right or wrong answers.
- There are no expected risks to participation. But you may find it awkward or uncomfortable to answer questions about your experience.
- There are no direct benefits to participating in this interview. But you may find it valuable to reflect on your experience.

We are interviewing many people in different roles to get a more complete picture of the program. You are the expert on your experience, and your opinions and thoughts are very important.

This interview is strictly confidential; meaning, information that identifies you will not be shared with anyone except our evaluation project team. We will never report your comments by name in any report

Your participation is voluntary. You may choose not to answer some of the questions or you may choose not to participate without penalty. You can stop the interview at any time for any reason. If you would like more information about the study or if you would like to withdraw from the study, you may contact the Principal Investigator, Dr. Amy DeGroff at 770-488-2415. If you have questions about your rights as a participant in this study, please contact CDC/ATSDR’s Acting Deputy Associate Director for Science at 1-800-584-8814. Leave a message with your name, phone number, and refer to CDC protocol #6569 and someone will call you back.

We would like to audiotape our conversation to assist with note taking and to make sure we accurately capture our discussion. Transcripts of audio files will be labeled with pseudonyms or fake names, and audio files and notes will be destroyed when the project is finished.

**Do you have any questions before we get started**? [ADDRESS ANY QUESTIONS AND THEN BEGIN.]

**Before we start our discussion, I would like to get verbal consent to proceed. Do you agree to participate in this interview?**

- Yes 🡪 Thank you. I confirm that you are willing to answer the questions in this discussion and will note your verbal consent. We would also like to record the conversation to make sure we don’t miss anything.
- No 🡪 *Thank participant for his or her time and end conversation.*

**Do I have your permission to turn on the audio recorder?**

- Yes 🡪 Thank you. *Turn on recorder.*
- No 🡪 Thank you. I will refrain from recording the session.

**Getting to know you**

1. I’m curious to hear about how you came to be a patient navigator for CRCSP. What initially appealed to you about the position? How long have you been a patient navigator with the program? What percent time is your position?
2. What other responsibilities and tasks, if any, do you have besides providing direct patient assistance?
3. Please tell me about your case mix, the patients you navigate. How are patients assigned to you? Do you serve a particular geographic area?

**Next, we’ll talk about the program model and teamwork.**

1. How was the idea of having a centralized, telephonic model of navigation developed? How did the model come about as a good fit here? Were other models considered?
2. If another state program were interested in replicating CRCSP, what would you tell them were the most essential elements of the program?

Probe: what core components must they have to replicate CRCSP’s program model?

1. Are there aspects of the program model that would be difficult to transfer to other settings?

probes: what is unique about this program or its context? Is the program affordable?

1. How does this program achieve such successful screening outcomes?

probe: What is important about the context in which you work?

probe: Are there other “intangible” things that help make the program work?)

1. Please describe how your job function relates to the other members of the CRCSP team. How does the team work together to accomplish programmatic results?
2. What barriers do you face in your job, providing PN?
3. What factors facilitate how you’re able to do your job?
4. Can you talk about key developmental milestones in implementing patient navigation---during start-up, once patient navigation first began, and at a more mature stage of implementation?

Probe: what challenges did you have to work through along the way & how did you do it?

1. How does your affiliation with Dartmouth Hitchcock Medical Center impact your work?

probe: Who is aware that you’re with Dartmouth, e.g., provider sites?

probe: How do you use Dartmouth’s reputation, if at all, to further your work?

1. How do you all think about, talk about, or plan for sustainability?

probe: Do you work with or engage partners to ensure sustainability?

probe: What aspects of the program are sustainable, with or without funding?

**Next, I’d like to discuss your general approach to navigating patients.**

1. Tell me more about your approach to patient empowerment. How and why was this approach chosen for this program?

probe: In what ways do you try to empower patients?

probe: How might a patient change as a result of your empowerment efforts?

1. Please tell me how you assess a patient’s level of motivation to undergo colonoscopy, either formally or informally. During navigation, do you use different strategies with patients who have different levels of motivation?
2. As best you know, what happens to patients who never enroll or get to the appointment-making step? How, if at all, have staff discussed trying to serve this especially hard to reach population?
3. Describe the range of navigation services you provide. For example, please describe a typical low and high-intensity patient.
4. How, if at all, do you vary your approach in dealing with patients based on their gender? Based on whether English is a first language?
5. What professional background, skills, competencies, and personal characteristics are important for a patient navigator?

Probe: Patient navigation has both “spirit” and “technique,” or is both art and science. Please describe important elements of both.

1. What type of training is necessary to do this job? Is clinical expertise necessary? Why or why not?

**Now, we’ll turn to how navigators work with systems and networks on behalf of patients.**

1. Providing patient navigation requires both a navigator and various systems. Please describe your role and how you interact with various systems.

Probe: how does the Catalyst database support your work?

Probe: With what other systems do you interact, e.g., google maps, language line?)

1. Please describe the network, or external contacts, for providing patient navigation services. How do you foster and nurture relationships with various agencies and partners? *(note: fostering networks is in the context of being a PN)*
2. *In your role as a PN,* please describe how, if at all, you’ve been able to make systems-level changes to promote colorectal screening.

Probe: have you been able to influence broader systems or provider groups, like GI practices, hospitals, or Dartmouth Medical Center?

Probe: Have you been able to promote quality screening more broadly than what happens as part of CRCSP?)

1. *FOR GAIL*, can you talk about how your other job responsibilities with systems change affect your navigation work?

Probe: do you approach it differently in some way?

1. Is there anything I didn’t ask about that you feel is important to mention?

**Wrap-Up**

As you know, in the upcoming weeks, we’d like to interview a sample of patients about their navigation experiences. We’ll contact you later by phone to discuss a few navigation cases that you’ll help us select and invite to participate. Once you get his/her permission for us to contact him/her, we will send him/her a letter in the mail explaining the purpose of our study. During the interview, we will ask the patient about his/her colonoscopy results and re-screening or surveillance interval. We will return just those data to you and ask you to verify the accuracy of recall. When you seek permission for us to contact him/her, please don’t alert their attention to this detail as we want to capture the typical recall experience.

**Thank you so much for your time today. Your insights will help us to better understand the patient navigation program.**

**[stop audio recorder]**

**STOP HERE**

**NOTE: will we audiotape this part?**

**Phone Follow-up with PNs on Specific Patients**

**Next we’ll talk about your work with helping 4-5 specific patients complete colonoscopy. Please feel free to refer to your patient notes in Catalyst. (Demographics and contact information will be gathered separately. Please refer to patients using a pseudonym or number.)**

***think about sampling frame & what we want to emphasize knowing. (Examples: particularly challenging cases, males/females, hard to motivate, low barrier/high barrier)***

**Patient Pseudonym: _______________**

1. How would you describe your relationship or experience with this patient? Did the nature of the relationship change over time or change at any specific points?
2. As you reflect on your work with this patient, were there high points and low points? Please describe a high point and a low point.
3. What was his/her major barrier to completing colonoscopy? How did you help resolve that? (Probe: What solutions did you offer or suggest? What other strategies did you use to help them complete their colonoscopy?)
4. How did you empower the patient? (Probe: How did you help him/her think about seeking healthcare differently?) What is your assessment of this patient’s level of empowerment?
5. Please tell me how you went about tailoring/providing culturally-appropriate services to this client.

**[repeat for each patient]**

**Thank you so much. We’ll follow-up with you by asking you to score the patients’ recall of the final results, including clinical findings and re-screening or surveillance interval.**
