# Supplementary material for: Assessing the implementation of a patient navigation intervention for colonoscopy screening
Source: BMC Health Serv Res. 2019 Nov 6;19:803. doi: 10.1186/s12913-019-4601-4 (PMC6833190; doi:10.1186/s12913-019-4601-4)
Supplement: Supplementary file 3 — Additional file 3. Interview guide used with endoscopists involved in the NHCRCSP. [file 12913_2019_4601_MOESM3_ESM.docx]

**Interview Guide for ENDOSCOPISTS (e.g., GIs)**

**Introduction and Informed Consent Statement**

Hi. My name is __________ with the Centers for Disease Control and Prevention. Thank you for giving us this opportunity to discuss your experiences with New Hampshire’s Patient Navigation for Colonoscopy Program. This should take no more than *60 minutes* of your time, and we’ll do our best to stay on track. Before we begin, let me explain the purpose of the study and your rights as a participant. Did you receive the informed consent form in the mail *[or by e-mail]*?

[*For in-person interviews, give one copy of the Informed Consent Form to the participant. Read the consent form as the participant follows along. Ask the participant if he/she has any questions about the study. After questions are answered, ask whether the participant would like to participate in the interview and, if so, ask the participant to sign the form. Next, ask if the participant gives permission to turn on the audio recorder and, if so, ask the participant to mark “Yes” where indicated. Collect the signed Informed Consent Form and give the participant a clean copy for his/her records. Proceed with the interview.]*

[*For telephone interviews, continue reading]*

In partnership with New Hampshire’s Colorectal Cancer Screening Program, managed by Dartmouth-Hitchcock Medical Center, the Centers for Disease Control and Prevention (CDC), Division of Cancer Prevention and Control, is conducting an evaluation of program impact. Simply stated, we want to understand how patient navigation can improve cancer screening through colonoscopy.

Let’s go over a few key points:

- This interview is not meant to evaluate you;
- Rather, it is meant to learn from you how patient navigation affects colorectal cancer screening. There are no right or wrong answers.
- There are no expected risks to participation. But you may find it awkward or uncomfortable to answer questions about your experience.
- There are no direct benefits to participating in this interview. But you may find it valuable to reflect on your experience.

We are interviewing many people in different roles to get a more complete picture of the program. You are the expert on your experience, and your opinions and thoughts are very important.

This interview is strictly confidential; meaning, information that identifies you will not be shared with anyone except our evaluation project team. We will never report your comments by name in any report.

Your participation is voluntary. You may choose not to answer some of the questions or you may choose not to participate without penalty. You can stop the interview at any time for any reason. If you would like more information about the study or if you would like to withdraw from the study, you may contact the Principal Investigator, Dr. Amy DeGroff at 770-488-2415. If you have questions about your rights as a participant in this study, please contact CDC/ATSDR’s Acting Deputy Associate Director for Science at 1-800-584-8814. Leave a message with your name, phone number, and refer to CDC protocol #6569 and someone will call you back.

We would like to audiotape our conversation to assist with note taking and to make sure we accurately capture our discussion. Transcripts of audio files will be labeled with pseudonyms or fake names, and audio files and notes will be destroyed when the project is finished.

**Do you have any questions before we get started**? [ADDRESS ANY QUESTIONS AND THEN BEGIN.]

**Before we start our discussion, I would like to get verbal consent to proceed. Do you agree to participate in this interview?**

- Yes 🡪 Thank you. I confirm that you are willing to answer the questions in this discussion and will note your verbal consent. We would also like to record the conversation to make sure we don’t miss anything.
- No 🡪 *Thank participant for his or her time and end conversation.*

**Do I have your permission to turn on the audio recorder?**

- Yes 🡪 Thank you. *Turn on recorder.*
- No 🡪 Thank you. I will refrain from recording the session.

*To begin, I’d like to ask about your background and role in this endoscopy practice…*

1. How long have you been performing colonoscopies? How long have you worked in this particular practice setting? Have you performed colonoscopies in other practice settings, health systems, or in other parts of the country?
2. How would you describe the patient population served by this practice? (e.g., rural, urban, low-income)

Next, I have a few questions about the CRCSP program…

1. Please describe who or what motivated you to participate in the CRCSP program.
2. From your point of view, what are the day to day challenges addressed by the CRCSP program’s **patient navigation** **component**? *[By patient navigation, we mean having the nurse navigators provide support to patients by telephone throughout the screening process.]*

probe: to your practice (e.g., decreasing no show rates)?

probe: to patients (e.g., improving adherence)?

probe: to improving exam quality (e.g., better bowel prep)?

1. What are the pros/cons of your participation in this program?
2. Would you recommend this program to other endoscopists? Why or why not?

Next, I’d like to talk about quality improvement…

1. How have you interacted with CRCSP staff, either with Dr. Lynn Butterly or the nurse navigators, around quality improvement efforts?
2. Have you changed anything about the way you do things as a result of your participation in this program?

Probe: in the way you perform colonoscopies? set re-screening/surveillance intervals? handle patient or PCP communication?

1. Have you noticed quality-related differences over time, from the beginning of your involvement with the program until now?

probe: are you able to detect any differences between those patients enrolled in CRCSP vs. those who aren’t?

probe: do you see better bowel prep among patients served by patient navigators?

probe: do you notice fewer no shows or cancellations?

Next, I have a few “big picture” questions about the program…

1. This program boasts remarkable colonoscopy adherence rates. Why, in general, do you think the program is so successful?
2. How important is clinical expertise for navigators? Could this program work with community health workers, for example? Why or why not?
3. This program serves multiple endoscopy sites from a central location with telephonic navigation. How would this program work outside the Dartmouth system?
4. What would it take to replicate the patient navigation program in a GI clinic setting? What key elements of the program would have to be replicated to make it work? Do you view this program as affordable?
5. Is there anything I didn’t ask about that you feel is important to mention?

**Thank you so much for your time today. Your insights will help us to better understand the patient navigation program.**

**[stop audio recorder]**
