# Supplementary material for: Assessing the implementation of a patient navigation intervention for colonoscopy screening
Source: BMC Health Serv Res. 2019 Nov 6;19:803. doi: 10.1186/s12913-019-4601-4 (PMC6833190; doi:10.1186/s12913-019-4601-4)
Supplement: Supplementary file 4 — Additional file 4. Interview guide used with practice administrators at endoscopy sites involved in the NHCRCSP. [file 12913_2019_4601_MOESM4_ESM.docx]

**Interview Guide for ENDOSCOPY Practice Administrators**

**Introduction and Informed Consent Statement**

Hi. My name is __________ with the Centers for Disease Control and Prevention. Thank you for giving us this opportunity to discuss your experiences with New Hampshire’s Patient Navigation for Colonoscopy Program. This should take no more than *60 minutes* of your time, and we’ll do our best to stay on track. Before we begin, let me explain the purpose of the study and your rights as a participant. Did you receive the informed consent form in the mail *[or by e-mail]*?

[*For in-person interviews, give one copy of the Informed Consent Form to the participant. Read the consent form as the participant follows along. Ask the participant if he/she has any questions about the study. After questions are answered, ask whether the participant would like to participate in the interview and, if so, ask the participant to sign the form. Next, ask if the participant gives permission to turn on the audio recorder and, if so, ask the participant to mark “Yes” where indicated. Collect the signed Informed Consent Form and give the participant a clean copy for his/her records. Proceed with the interview.]*

[*For telephone interviews, continue reading]*

In partnership with New Hampshire’s Colorectal Cancer Screening Program, managed by Dartmouth-Hitchcock Medical Center, the Centers for Disease Control and Prevention (CDC), Division of Cancer Prevention and Control, is conducting an evaluation of program impact. Simply stated, we want to understand how patient navigation can improve cancer screening through colonoscopy.

Let’s go over a few key points:

- This interview is not meant to evaluate you;
- Rather, it is meant to learn from you how patient navigation affects colorectal cancer screening. There are no right or wrong answers.
- There are no expected risks to participation. But you may find it awkward or uncomfortable to answer questions about your experience.
- There are no direct benefits to participating in this interview. But you may find it valuable to reflect on your experience.

We are interviewing many people in different roles to get a more complete picture of the program. You are the expert on your experience, and your opinions and thoughts are very important.

This interview is strictly confidential; meaning, information that identifies you will not be shared with anyone except our evaluation project team. We will never report your comments by name in any report.

Your participation is voluntary. You may choose not to answer some of the questions or you may choose not to participate without penalty. You can stop the interview at any time for any reason. If you would like more information about the study or if you would like to withdraw from the study, you may contact the Principal Investigator, Dr. Amy DeGroff at 770-488-2415. If you have questions about your rights as a participant in this study, please contact CDC/ATSDR’s Acting Deputy Associate Director for Science at 1-800-584-8814. Leave a message with your name, phone number, and refer to CDC protocol #6569 and someone will call you back.

We would like to audiotape our conversation to assist with note taking and to make sure we accurately capture our discussion. Transcripts of audio files will be labeled with pseudonyms or fake names, and audio files and notes will be destroyed when the project is finished.

**Do you have any questions before we get started**? [ADDRESS ANY QUESTIONS AND THEN BEGIN.]

**Before we start our discussion, I would like to get verbal consent to proceed. Do you agree to participate in this interview?**

- Yes 🡪 Thank you. I confirm that you are willing to answer the questions in this discussion and will note your verbal consent. We would also like to record the conversation to make sure we don’t miss anything.
- No 🡪 *Thank participant for his or her time and end conversation.*

**Do I have your permission to turn on the audio recorder?**

- Yes 🡪 Thank you. *Turn on recorder.*
- No 🡪 Thank you. I will refrain from recording the session.

To get started….

1. Tell me about your role as a practice administrator. What are your main responsibilities? What kinds of things do you do to help implement CRCSP here?

probe: in what ways do you interact with the patient navigators?

1. Please describe how or why your practice became involved with this program.

probe: do you recall when you were first approached about participating? Please tell me about that.

Next, I have a few questions about the patient navigation component of the CRCSP program…

1. What does patient navigation do from your perspective? Does it help alleviate a specific problem in your practice? or with patients?
2. I want to understand each step in the process for patients, from initial referral to communicating the colonoscopy results. Please walk me through the general process for a typical patient. And, then, walk me through the process for a CRCSP patient. What’s different about the process for patients, and for you or your staff? (*For Clinic A*, have there been any changes in procedures since July 2012?)
3. How does having patient navigation with CRCSP streamline or add complexity to existing procedures or processes?
4. Your participation in this program has costs & benefits. Can you describe those, as well as how you view the return on investment?
5. What feedback (reports, data) do you receive about your clinic’s participation? Do you perform any analyses yourself about the costs/benefits to your participation?
6. Have you changed anything about your practice in general as a result of your involvement with CRCSP? Are there any specific lessons or “a-ha” moments you can point to?
7. Is there anything that surprised you about how this program works?

Next, I have a few “big picture” questions about the program…

1. CRCSP has achieved high colonoscopy adherence rates. Why does this program work so well?
2. How important is clinical expertise among the patient navigators to how this program works? Could it work with community health workers rather than nurses? Why or why not?
3. Why does the telephonic model work so well here, having a centralized location serving multiple clinics?
4. What would it take to replicate the patient navigation program in a GI clinic setting? Do you view this as an affordable program?
5. Is there anything I didn’t ask about that you feel is important to mention?

**Thank you so much for your time today. Your insights will help us to better understand the patient navigation program.**

**[stop audio recorder]**
