# Supplementary material for: Assessing the implementation of a patient navigation intervention for colonoscopy screening
Source: BMC Health Serv Res. 2019 Nov 6;19:803. doi: 10.1186/s12913-019-4601-4 (PMC6833190; doi:10.1186/s12913-019-4601-4)
Supplement: Supplementary file 5 — Additional file 5. Interview guides used with primary care providers involved in the NHCRCSP. [file 12913_2019_4601_MOESM5_ESM.docx]

**Interview Guide for PRIMARY CARE PHYSICIANS (PCPs)**

**Introduction and Informed Consent Statement**

Hi. My name is __________ with the Centers for Disease Control and Prevention. Thank you for giving us this opportunity to discuss your experiences with New Hampshire’s Patient Navigation for Colonoscopy Program. This should take no more than *60 minutes* of your time, and we’ll do our best to stay on track. Before we begin, let me explain the purpose of the study and your rights as a participant. Did you receive the informed consent form in the mail *[or by e-mail]*?

[*For in-person interviews, give one copy of the Informed Consent Form to the participant. Read the consent form as the participant follows along. Ask the participant if he/she has any questions about the study. After questions are answered, ask whether the participant would like to participate in the interview and, if so, ask the participant to sign the form. Next, ask if the participant gives permission to turn on the audio recorder and, if so, ask the participant to mark “Yes” where indicated. Collect the signed Informed Consent Form and give the participant a clean copy for his/her records. Proceed with the interview.]*

[*For telephone interviews, continue reading]*

In partnership with New Hampshire’s Colorectal Cancer Screening Program, managed by Dartmouth-Hitchcock Medical Center, the Centers for Disease Control and Prevention (CDC), Division of Cancer Prevention and Control, is conducting an evaluation of program impact. Simply stated, we want to understand how patient navigation can improve cancer screening through colonoscopy.

Let’s go over a few key points:

- This interview is not meant to evaluate you;
- Rather, it is meant to learn from you how patient navigation affects colorectal cancer screening.
- There are no right or wrong answers.
- There are no expected risks to participation. But you may find it awkward or uncomfortable to answer questions about your experience.
- There are no direct benefits to participating in this interview. But you may find it valuable to reflect on your experience.

We are interviewing many people in different roles to get a more complete picture of the program. You are the expert on your experience, and your opinions and thoughts are very important.

This interview is strictly confidential; meaning, information that identifies you will not be shared with anyone except our evaluation project team. We will never report your comments by name in any report.

Your participation is voluntary. You may choose not to answer some of the questions or you may choose not to participate without penalty. You can stop the interview at any time for any reason. If you would like more information about the study or if you would like to withdraw from the study, you may contact the Principal Investigator, Dr. Amy DeGroff at 770-488-2415. If you have questions about your rights as a participant in this study, please contact CDC/ATSDR’s Acting Deputy Associate Director for Science at 1-800-584-8814. Leave a message with your name, phone number, and refer to CDC protocol #6569 and someone will call you back.

We would like to audiotape our conversation to assist with note taking and to make sure we accurately capture our discussion. Transcripts of audio files will be labeled with pseudonyms or fake names, and audio files and notes will be destroyed when the project is finished.

**Do you have any questions before we get started**? [ADDRESS ANY QUESTIONS AND THEN BEGIN.]

**Before we start our discussion, I would like to get verbal consent to proceed. Do you agree to participate in this interview?**

- Yes 🡪 Thank you. I confirm that you are willing to answer the questions in this discussion and will note your verbal consent. We would also like to record the conversation to make sure we don’t miss anything.
- No 🡪 *Thank participant for his or her time and end conversation.*

**Do I have your permission to turn on the audio recorder?**

- Yes 🡪 Thank you. *Turn on recorder.*
- No 🡪 Thank you. I will refrain from recording the session.

1. I’d like to begin by hearing more about how you got started referring patients to this program.

probe: How did you hear about the CRCSP program?

probe: What did you know about the program before you got started?

1. How do you decide which patients to refer to CRCSP?

probe: Who stands to benefit most from this program?

probe: Which patients are a fit with this program?

*Next, I’d like to ask you about patient navigation.*

*Are you familiar with CRCSP’s patient navigation component? [*If not*, CRCSP has 2 nurse navigators who support patients by phone throughout the colonoscopy process..for example, navigators provide education about the procedure, ensure the patient has an escort on the day of the procedure, & make sure the patients have received and understand their results. ]*

1. From your point of view, what are the major benefits of **patient navigation** for colonoscopy, both to primary care physicians and to patients?

probe: what problem does patient navigation solve?

probe: How, if at all, does patient navigation make connections between patients and PCPs?

probe: How, if at all, does patient navigation make connections between PCPs and endoscopists?

1. From your perspective, are there any downsides to patient navigation? (*example downsides: additional communication burden, expenses*)

*Now, we’ll turn to specifics about how the program operates….*

1. Please describe the typical communication loop for your patients, in general, when you refer them out for colonoscopy, and how that may differ from your patients enrolled in CRCSP.

Probe: Do you know what happens after you refer them for a colonoscopy?

Probe: Do you typically receive their colonoscopy results?

1. Among your patients, do you notice any other differences between those patients enrolled in CRCSP vs. those who aren’t?

Probe: For example, are CRCSP patients more informed about their screening results?

1. What feedback do your patients provide you about the program? What feedback does the program provide you about your patients?
2. Has the program influenced any changes in how you provide care to your patients around colorectal screening? (*note: may have to redirect toward PN-related issues*)
3. Is there anything you’d like to change about the program?

*To wrap up, I have a few “big picture” questions about the program….*

1. The program has impressive colonoscopy adherence rates. From what you know about how the program operates, why do you think it’s so effective?
2. Some patient navigation program models use community health workers, rather than nurses. Could this program work with CHWs—why or why not?

probe: is clinical expertise important to how the program works? Please explain why.

1. This program serves multiple clinics from a central location using telephonic navigation. Why does this work so well here, in NH? With Dartmouth-Hitchcock Medical Center? What would it take to replicate this program somewhere else? Is this program affordable?
2. Is there anything else we haven’t talked about, that you feel is important to mention?

**Thank you so much for your time today. Your insights will help us to better understand the patient navigation program.**

**[stop audio recorder]**
