# Supplementary material for: Assessing the implementation of a patient navigation intervention for colonoscopy screening
Source: BMC Health Serv Res. 2019 Nov 6;19:803. doi: 10.1186/s12913-019-4601-4 (PMC6833190; doi:10.1186/s12913-019-4601-4)
Supplement: Supplementary file 6 — Additional file 6. Interview guides used with partner organization staff involved in the NHCRCSP. [file 12913_2019_4601_MOESM6_ESM.docx]

**Interview Guide for Stakeholders**

**Introduction and Informed Consent Statement**

Hi. My name is __________ with the Centers for Disease Control and Prevention. Thank you for giving us this opportunity to discuss your experiences with New Hampshire’s Patient Navigation for Colonoscopy Program. This should take no more than [*30 minutes, 60 minutes, or 90 minutes, depending on role]* of your time, and we’ll do our best to stay on track. Before we begin, let me explain the purpose of the study and your rights as a participant. Did you receive the informed consent form in the mail *[or by e-mail]*?

[*For in-person interviews, give one copy of the Informed Consent Form to the participant. Read the consent form as the participant follows along. Ask the participant if he/she has any questions about the study. After questions are answered, ask whether the participant would like to participate in the interview and, if so, ask the participant to sign the form. Next, ask if the participant gives permission to turn on the audio recorder and, if so, ask the participant to mark “Yes” where indicated. Collect the signed Informed Consent Form and give the participant a clean copy for his/her records. Proceed with the interview.]*

[*For telephone interviews, continue reading]*

In partnership with New Hampshire’s Colorectal Cancer Screening Program, managed by Dartmouth-Hitchcock Medical Center, the Centers for Disease Control and Prevention (CDC), Division of Cancer Prevention and Control, is conducting an evaluation of program impact. Simply stated, we want to understand how patient navigation can improve cancer screening through colonoscopy.

Let’s go over a few key points:

- This interview is not meant to evaluate you;
- Rather, it is meant to learn from you how patient navigation affects colorectal cancer screening. There are no right or wrong answers.
- There are no expected risks to participation. But you may find it awkward or uncomfortable to answer questions about your experience.
- There are no direct benefits to participating in this interview. But you may find it valuable to reflect on your experience.

We are interviewing many people in different roles to get a more complete picture of the program. You are the expert on your experience, and your opinions and thoughts are very important.

This interview is strictly confidential; meaning, information that identifies you will not be shared with anyone except our evaluation project team. We will never report your comments by name in any report.

Your participation is voluntary. You may choose not to answer some of the questions or you may choose not to participate without penalty. You can stop the interview at any time for any reason. If you would like more information about the study or if you would like to withdraw from the study, you may contact the Principal Investigator, Dr. Amy DeGroff at 770-488-2415. If you have questions about your rights as a participant in this study, please contact CDC/ATSDR’s Acting Deputy Associate Director for Science at 1-800-584-8814. Leave a message with your name, phone number, and refer to CDC protocol #6569 and someone will call you back.

We would like to audiotape our conversation to assist with note taking and to make sure we accurately capture our discussion. Transcripts of audio files will be labeled with pseudonyms or fake names, and audio files and notes will be destroyed when the project is finished.

**Do you have any questions before we get started**? [ADDRESS ANY QUESTIONS AND THEN BEGIN.]

**Before we start our discussion, I would like to get verbal consent to proceed. Do you agree to participate in this interview?**

- Yes 🡪 Thank you. I confirm that you are willing to answer the questions in this discussion and will note your verbal consent. We would also like to record the conversation to make sure we don’t miss anything.
- No 🡪 *Thank participant for his or her time and end conversation.*

**Do I have your permission to turn on the audio recorder?**

- Yes 🡪 Thank you. *Turn on recorder.*
- No 🡪 Thank you. I will refrain from recording the session.

1. Please describe your involvement with the New Hampshire Colorectal Cancer Screening Program (CRCSP). Are you familiar with how they conduct Patient Navigation for Colonoscopy?
2. What are your impressions of the program? Why do you think this program is, or is not, so successful?
3. What factors facilitate program implementation?

Probe: There are numerous facets to the program---the program structure, staff, partnerships, Dartmouth’s reputation & clinical relationships—what aspects contribute most to how the program works?

1. **For HD staff**: Please tell me about the state health department’s decision to use Dartmouth as its bonafide agent for this FOA. Why were they particularly well-suited to implement this program?
2. What challenges does the program face? Can you talk about the program’s key developmental milestones during start-up, once patient navigation first began, and at a more mature stage of implementation?
3. Who or what are essential partners to help support this program? & why?
4. What are main benefits of this patient navigation program?

probe: What have you learned about the importance of patient navigation?

probe: What is the significance---to state/region, to your organization, to public health, to advancing population-based screening?

probe: How does this program address health disparities?

1. Did anything about how this program works surprise you?
2. Could this model, that is, nurses providing statewide telephonic navigation, work in other settings (e.g., GI practices, public health depts.)? Why or why not?
3. **State HD**: what would it take to replicate this program within public health departments?
4. Is clinical expertise necessary for navigators? Why or why not?
5. How affordable is the program? If the program had fewer resources, what are the most essential elements to the program?
6. How can the program sustain its efforts over time?
7. Is there anything I didn’t ask about that you feel is important to mention?

**Thank you so much for your time today. Your insights will help us to better understand the patient navigation program.**

**[stop audio recorder]**
